# Ecological Network Analysis for a Low-Carbon and High-Tech Industrial Park

**DOI:** 10.1100/2012/305474

**Published:** 2012-12-18

**Authors:** Yi Lu, Meirong Su, Gengyuan Liu, Bin Chen, Shiyi Zhou, Meiming Jiang

**Affiliations:** ^1^State Key Joint Laboratory of Environmental Simulation and Pollution Control, School of Environment, Beijing Normal University, Beijing 100875, China; ^2^Beijing Development Area Co. Ltd, Beijing 100176, China

## Abstract

Industrial sector is one of the indispensable contributors in global warming. Even if the occurrence of ecoindustrial parks (EIPs) seems to be a good improvement in saving ecological crises, there is still a lack of definitional clarity and in-depth researches on low-carbon industrial parks. In order to reveal the processes of carbon metabolism in a low-carbon high-tech industrial park, we selected Beijing Development Area (BDA) International Business Park in Beijing, China as case study, establishing a seven-compartment- model low-carbon metabolic network based on the methodology of Ecological Network Analysis (ENA). Integrating the Network Utility Analysis (NUA), Network Control Analysis (NCA), and system-wide indicators, we compartmentalized system sectors into ecological structure and analyzed dependence and control degree based on carbon metabolism. The results suggest that indirect flows reveal more mutuality and exploitation relation between system compartments and they are prone to positive sides for the stability of the whole system. The ecological structure develops well as an approximate pyramidal structure, and the carbon metabolism of BDA proves self-mutualistic and sustainable. Construction and waste management were found to be two active sectors impacting carbon metabolism, which was mainly regulated by internal and external environment.

## 1. Introduction

The study of ecoindustrial parks (EIPs) has assumed great deal of importance within the past ten to fifteen years. One of the best definitions of an EIP has been provided by the UESPA, which was stated as “*a community of manufacturing and service businesses seeking enhanced environmental and economic performance by collaborating in the management of environmental and reuse issues. By working together the community of businesses seeks a collective benefit that is greater than the sum of the individual benefits each company would realize if it optimized its individual performance only*” [1]. This definition mainly aims to close material cycles in the industrial chain and considers the entire life cycle from raw material production to product consumption and waste management [2].

Despite cooperation between companies to find win-win solutions [3], from the traditional perspective of EIPs, one of their strong points is basically focused on the structural planning and functional utility of industrial recycling. It means that a better design or running of EIPs' cycle have arisen to reduce waste management and disposal costs, extract with cheaper materials and energy, and gain benefits from residues [4]. However, in the more furious conflict between development of economic society and recovery of polluted ecosystem, grimmer situation and stricter requirements will be put forward. Traditional definition of EIPs cannot adapt to the social development better, especially when the whole world is paying more attention on climate change.

Climate change announces a fast global socioeconomic transition, but nobody could predict the ultimate results of the next industrial revolution. Obviously, business and government play a key role in promoting or destroying the revolution. It is undeniable that climate change affects both natural ecosystems and human societies [5–7]. Taking climate change into consideration, EIPs can be classified into green industry parks and integrated ecoindustry parks. Therein, green industry park was defined as “*a range of enterprises that use cleaner production technologies, process much of their waste and/or reduce the emissions of greenhouse gases, in situ*” [8]. In Belgium, green industry park is embodied as the “carbon neutral industrial parks,” an initiative of the Flemish region, which brings in the carbon footprint of companies into the quality requirements for industrial parks to enforce carbon emission reduction and sustainable energy policy measures [7]. And some studies have suggested that it is practical to implement climate change through industrial symbiosis [9, 10] and energy innovation in EIPs [11].

An industrial ecosystem is constructed with the flows of matter, nutrients, energy, and carbon [12]. As a necessary part of industrial flows, some scientists make great contribution to stretch out the carbon cycle in different industrial ecosystem. Korhonen et al. considered the material (including carbon flow) and energy flows of forest ecosystem in Finland [12]. Liu et al. provided an outline of energy-based greenhouse gas emissions inventory in Suzhou Industrial Park, China [13]. Norstebo et al. and Midthun et al. both analyzed the taxation of CO_2_ emissions and carbon capture in a case study of an extension of an EIP in Norway [14, 15]. Munir et al. presented a newly developed carbon emission Pinch Analysis technique for achieving holistic minimum carbon targets in EIPs [16]. Besides, there is still a lack of speciality of the system-wide carbon emission in an EIP.

EIPs originate from three major concepts, namely, scilicet industrial ecology, biological ecology, and the spatial perspectives based on landscape ecology [17]. In the second field of EIPs, scientists have tried to seek the inner-mimicry from biological individuals with industrial unit to natural ecosystem with industrial system. It is a metaphor running through the biological-industrial process, structure, and function. Hence the concept of industrial metabolism is first established by Ayres, which is defined as “*the whole integrated collection of physical processes that convert raw materials and energy, plus labor, into finished products and wastes*” [18, 19]. The summary of industrial metabolism is analyzing the whole material flows, extracting all possible emission sources, and assessing the influences within these flows [20]. After that, Graedel shows a good example in using statistical indexes of food web from natural ecosystems for reference in his evaluations of EIPs [21].

Nowadays, diverse analysis tools are used in the research of EIPs, which can be classified into two main trends of methodology. One of them is generally based on the inventorying of life-cycle ecological and economic input-output flows, including material flow analysis [3, 22], input-output analysis [23], life-cycle assessment [24], and structure and network analysis based on industrial metabolism [20, 25, 26]. The other method is concerned with the idea of the available solar energy.

Ecological Network Analysis (ENA) is a general metabolism-based analytical tool for studying the system connectivity and for quantifying and qualifying direct and indirect ecological flows in the system [27]. In general, the key concepts of ENA are behavior, structure, and function of a system [27]. An EIP can be extracted as a symbiotic network, where many material and energy flows link diverse compartments in ensuring a smooth running of industrial processes (both of the components' mutual interactions in integral environment and the passing relationship between integral and external environment) and system functioning. So it is appropriate to introduce ENA into EIP study. However, present attempts in seeking carbon metabolism in EIPs based on the network view are still a blank space, and challenges include defining what low-carbon high-tech industrial park is (especially in what ‘‘low-carbon” could be defined), understanding why these parks could reduce more carbon emission, as well as seeking to use an accessible tool for metabolic structure and functioning study. The adoption of ENA may provide a feasible prospect in resolving the latter two issues by evaluating the metabolic intensities, processes, structure, and control of carbon emissions.

In China, there exists a conflict among economic development, shortage of natural resource, and serious pollution suggested by the impetus for developing EIPs [28]. Hence developing high-tech industrial parks has been a promising trend of EIPs' development. These EIPs generally assemble high-tech business located in the upstream of industrial chain, such as companies in the domains of intellective, R & D, design, or head office. BDA International Business Park (BDA) is situated in Beijing High-Tech Industrial Park, which is characterized as its graceful ecological environment, low energy consumption, low carbon emission intensity, and amassing of high-tech business. As a feasible trial in developing low-carbon industrial parks, BDA covers a landscape area of 0.1735 km^2^ and a construction area of 0.336 km^2^. The park consists of 34 separate office buildings where 159 high-tech companies have been stationed in total. It is worth emphasizing that BDA is the first EIP considering the low-carbonic concept in its design in Beijing. This study tries to present an ENA-based methodology for carbon metabolism in low-carbon high-tech industrial parks and selects BDA as a case study for promoting carbon reduction in EIPs.

## 2. Materials and Methods

### 2.1. Ecological Network Model for Low-Carbon High-Tech Industrial Park

The essence of ecological network model is a transmitting network for materials and energy, which includes both of the components' mutual interactions in integral environment and the passing relationship between integral and external environment. For this sake, establishing the reasonable system boundary and making sure of limiting factors should be the necessary step for the ecological network model.

**Figure 1:**
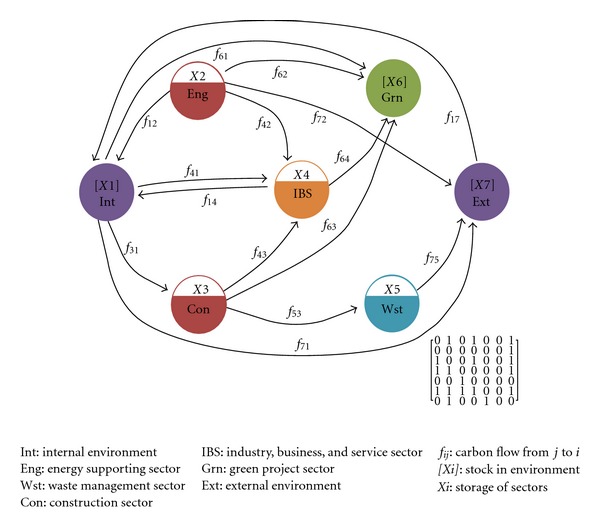
Metabolic network model for carbon metabolism of BDA International Business Park. The matrix at the right bottom is the adjacency matrix A of the model, where A = [*a*_*ij*_]. If there exists a carbon flow from compartment *j* to *i*, *a*_*ij*_ = 1, else *a*_*ij*_ = 0.

Even though EIPs are mainly artificially controlled, both artificial and natural processes of parks' carbon fluxes should be taken into account in the network model. The system boundary does not just coincide with the administrative boundaries, but a virtual boundary that contains metabolic processes links both inside and outside of the park. Taking carbon metabolic processes and their relationship through different compartments within the virtual boundary, a metabolic network model for low-carbon high-tech industrial parks (we might call it as Low-Carbon Metabolic Network (LCMN) as well) is established for tracking carbon flows within an low-carbon park ecosystem (Figure 1). In the LCMN, it embraces seven individual compartments: energy supporting sector (Eng), construction sector (Con), industry, business and service sector (IBS), waste management sector (Wst), green project sector (Grn), internal environment (Int), and external environment (Ext). Diverse carbon fluxes running through these compartments are identified and characterized as the compartmental interactions within LCMN, including the flows within Eng, Con, IBS, and Wst in terms of exchanging materials (both goods and wastes) and energy, flows between the park and its external environment, and the natural carbon exchange of the park. Besides, the flow of goods transporting and transport fuel induced by BDA (within and outside the park) were also involved.

### 2.2. Ecological Network Analysis

#### 2.2.1. Network Utility Analysis (NUA)

“Utility” is an economic conception similar to “efficiency.” Since Patten [29, 30] firstly introduced the concept of NUA, it has mainly been applied to indicate both qualitative and quantitative exchange-based relationships between different compartments of a network system [27]. The relation forms within components are in variety, where one of the simplest ideas is sorted as direct and indirect (similar as a series of consequent direct transfer). In NUA, direct and integral relationships are expressed by a direct utility matrix **D** and dimensionless integral utility intensity matrix **U**, respectively. Matrix **D** = [*d*_*ij*_] illustrates the relative strength of direct input and output control in the network [27]. *d*_*ij*_ represents the direct interactions between compartment *i* and *j*, which can be expressed as

(1) $$\begin{array}{c} d_{ij}=\frac{\left(f_{ij}-f_{ji}\right)}{T_{i}}, \end{array}$$

where *f*_*ij*_ represents the metabolic flow (e.g., carbon flow) from compartment *j* to *i*; *T*_*i*_ is the sum of input or output flows for the *i*th compartment at the steady state. Then, whole-system, integral, utility-based relations are given by considering all the indirect influences in the network carried by the higher-order interactions [31]. Distinguished with the direct utility matrix **D**, integral utility intensity matrix **U** contributes to reveal the strength of the entire network organization. Compared with the sum of elements between the direct and indirect matrix, it could often find a greater contribution from indirect processes than from the direct one [27]. For revealing the net utility of each compartment to make use of materials along different-step pathways, matrix **U** is computed as

(2) $$\begin{array}{c} U=D^{0}+D^{1}+D^{2}+\cdots +D^{m}={\left(I-D\right)}^{-1}, \end{array}$$

where **U** shows the utilities conveyed by pathways in different lengths 1,2,…, *m* (*m* is the total number of compartments, *m* ≥ 2); the identity matrix **I** shows the self-feedback of flows through each compartment; the matrix **D** ^1^ reflects the direct interactions between components; **D** ^*m*^ represents the indirect relations between components along *m*-length-pathway. In the view of common network analysis indirect interactions **D** ^*k*^  (2 ≤ *k* ≤ *m*) means specific materials convey via relative longer pathways greater than length one, which can be verified by taking the higher order powers of **D**, for example, **D** ^2^ gives utilities conveyed along two-step pathways, **D** ^3^ is along three-step pathways, and so on [31].

In NUA, Sign *D* and Sign *U* are introduced as two sign matrices of **D** and **U** in order to reveal the mutualism relationship between components. Compared with Sign *D*, Sign *U* gives a deeper perspective in revealing potential connections between each component. Referring to the gain (+), loss (−), or neutrality (0), interactions can be calculated by two objects: (+, +) stands for mutualistic condition, (+,  −) for exploitation condition, (−,  +) for exploited condition, (−,  −) for competition, and (0, 0) for neutrality [31]. In these two matrices, the sign changing would affect the interactions between components, and then affect the network structure.

Fath and Patten [27] then investigated network synergism (also known as mutualism), another NUA property, to convey that positive utility was more than negative utility in quantity. There are two ways for testing the network synergism: one of them can be quantified by total utility in the dimensional utility matrix, while another one is revealed by the ratio of positive to negative utility in the network system [27]. Hence, at the level of entire system, network mutualism index (MI) and synergism index (SI) are adapted to show the fitness of the whole-system [32–34]. MI reflects the ratio of the number of positive and negative signs in the Sign*U*. While SI quantifies the total magnitude of the positive and negative utilities, which assess the mutualistic condition of a system in slightly different angles [35]. If MI is greater than one, or SI is greater than zero, the system mutualism could occur [27, 29, 30]. MI and SI are computed as

(3) $$\begin{array}{c} \text{MI}=\frac{\text{Sign}U\left(+\right)}{\text{Sign}U\left(-\right)}, \end{array}$$

(4) $$\begin{array}{c} \text{SI}=\sum_{j=1}^{n}\;\sum_{i=1}^{n}u_{ij}, \end{array}$$

where

(5) SignU(+)=∑ijmax⁡(Sign(uij),0),SignU(−)=∑ij−min⁡(Sign(uij),0).

#### 2.2.2. Network Control Analysis (NCA)

Patten [36] introduced NEA-based measures of control or dominance by using the input and output environ concept to develop a control matrix [37, 38]. Network control is based on a pair of integral flow through network flow analysis, which indicates the control from system compartments in the configuration of the whole system [35]. And network flow analysis is predicated on a conceptual flow model of a system, revealing both the structure and function of the system [39].

Be similar as the direct utility matrix *D* and integral utility matrix *U*, in control analysis (or flow analysis), flow interactions can also be divided into direct and integral (including initial input, direct, and indirect interactions) ones. Matrix **G** is the direct interaction matrix, giving the functional influences due to all paths of lengths commensurate with the power. While matrix **N** shows the indirect interaction, summing the infinite power series of the direct interaction matrix [39].

For the output environ, from the generating or flow-forward transfer efficiencies and the receiving or flow-backward transfer efficiencies **G** = [*g*_*ij*_] and **G**′ = [*g*_*ij*_′], dimensionless integral output and input flow intensity matrices **N** = [*n*_*ij*_] and **N**′ = [*n*_*ij*_′] can be computed as

(6) $$\begin{array}{c} N=\left[n_{ij}\right]={\left(I-G\right)}^{-1},\;\;N^{\prime }=\left[n_{ij}^{\prime }\right]={\left(I-G^{\prime }\right)}^{-1}, \end{array}$$

where *g*_*ij*_ = *f*_*ij*_/*T*_*j*_, it shows the nondimensional, output-oriented, intercompartmental flows; if *g*_*ij*_′ = *f*_*ij*_/*T*_*i*_, it shows the input-oriented, intercompartmental flows [32, 38]. So two distributed control metrics based on these could be established to reflect the control and dependence condition, which are control allocation (CA) and dependence allocation (DA)

(7) CA=[caij]≡{nij−nij′>0,  caij=nij−nij′∑i=1mnij−nij′nij−nij′≤0,  caij=0,

(8) DA=[daij]≡{nij−nij′>0,  daij=nij−nij′∑i=1mnij−nij′nij−nij′≤0,  daij=0,

where 0 ≤ *da*_*ij*_, *ca*_*ij*_ ≤ 1. By definition both CA and DA are calculated by the difference of two pairwise integral flows (i.e., *n*_*ij*_ and *n*_*ij*_′). *ca*_*ij*_ reflects the degree that compartment *j* controls compartment *i* based on the controller's output environ, while *da*_*ij*_ indicates the degree that compartment *j* is dependent on compartment *i* from the observer's input environ.

Based on the network control and dependence formulation, the system-wide control condition can be revealed by the system control index (CI). CI combines control degree with dependence degree, and thus it indicates the control utility and organization capability of the whole system and can be employed to index the self-regulation of system metabolism [35] as follows:

(9) CI≡∑j=1m∑i=1mcaij+∑j=1m∑i=1mdaijm2.

#### 2.2.3. System-Wide Indicators

For the purpose of giving an overall perspective on metabolic performance of industrial park and contributing to design a both sustainable and low-carbonic park, it is necessary to define a set of indicators in addressing the system performance of the MN. Some of these indicators have already been introduced by NUA and NCA as above, while others were extracted from other researches of ENA [28]. Each indicator reflects a facet of carbon metabolism in LCMN for BDA. The formulations and short description of the whole-system indicators were illustrated (Table 1).

### 2.3. Data Source

The metabolism data sources were extracted from construction and operation data of BDA which were all calculated based on the IPCC recommended method and life cycle analysis. These data originated from investigations and calculation into carbon composition of artificial activities, raw materials' transportation, the relationships between these flows and stocks, and also within anthropogenic-natural processes.

## 3. Results

### 3.1. Ecological Structure of Carbon Emissions

Carbon fluxes between two compartments within LCMN of BDA are listed in Table 2. The result shows that Con (11.2%), Eng (8.2%), and Wst (8.2%) are three major carbon donors providing carbon to Con (41.4%) and Grn (24.5%), two major carbon accepters, in the form of materials, wastes, fossil fuels and machinery, and so forth. Diverse carbon exchanges between these major donors and accepters make a great contribution to support the operation of the park. Yet Eng and Wst's (both 8.2%) contribution in accepting carbon are not as great as their superior performance on the supply side. Compared with these major sectors, IBS as one of the most inactive sectors is both inferior in supplying and accepting carbon (3.9% and 6.7% resp.,). The ultimate suppliers and recipients of carbon are Int and Ext. Among which, both supplying and receipting of Int are not as much as other sectors (3.8% and 4.3% resp.,) showing a weak effect on the LCMN of BDA; Ext has a better performance in supplying (64.7%) than accepting carbon (8.9%), indicating the BDA park may be more dependent on the supply of the external environment. The biggest carbon emissions are from Eng to Int (3046 t CO_2_-eq) and Wst to Ext (17547 t CO_2_-eq). And IBS (6001 t CO_2_-eq) and Grn (2176 t CO_2_-eq) are two major sectors obtaining carbon from internal environment, which are much more than Con (13 t CO_2_-eq). Similarly, Con (82509 t CO_2_-eq), Grn (39620 t CO_2_-eq), and Eng (12796 t CO_2_-eq) are all extracting carbon greatly from the external environment, revealing that these three main sectors are more dependent on external supply again. The carbon emission (from Wst to Ext, 8.2%), extraction (from Ext to Con, 38.6%), and processing (from Con to Wst, 8.2%) are the most active and significant processes. Overall, the total carbon through flow of BDA is 213670 t CO_2_-eq.

**Figure 2:**
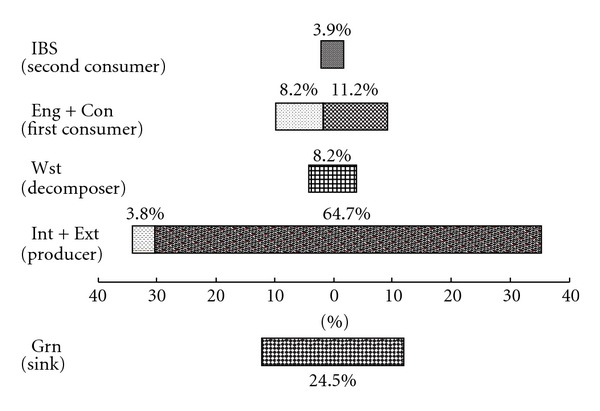
Ecological structure of carbon metabolism in BDA.

The proportion of carbon flows within each sector can reflect the ecological structure of the carbon metabolism in BDA, which forms an approximate pyramidal shape (Figure 2). In natural ecosystem, the pyramidal trophic structure based on the food web is one of the most stable structures leading an ordered and healthy ecosystem. In this sense, the carbon metabolic system in BDA is also in stable condition relatively in the role of producer (Int and Ext), decomposer (Wst), first consumer (Eng and Con), and second consumer (IBS). Besides, as a little deficiency, the decomposer cannot take full advantage thanks to the limit of waste managing technology. If we enhance the role of decomposers in the BDA's carbon metabolic network, the utilization efficiency and system stability of carbon metabolism will be improved.

Reading from left to right, the values are the total carbon inputs or outputs (Grn fits the output value) of compartments. Producer: supplying distal carbon into BDA system; decomposer: releasing carbon for producers' reuse by decomposition of the waste; first consumer: carbon agents that transfer carbon from natural environment to human society; second consumer: anthropogenic using processed carbon resources from first consumers by processes of creating products or utilizing energy; sink: eliminating carbon through photosynthesis of green trees, it does not belong to the trophic structure.

### 3.2. Network Mutual Relationships

Table 3, respectively, shows the direct mutual relationships (in the matrix Sign*D*) and integral interactions (in the matrix Sign*U*) between such compartments of LCMN in BDA. It is apparently that the positive/negative signs in the matrix Sign*U* sometimes vary from those in the matrix Sign*D*, which implies that both quantities and qualities of the integral relationships could alter compared with those direct ones [27]. By comparing the relation changes between direct and integral utility matrix, it is obvious that circumstances of no changes occur most frequently, that is, the relationship of IBS-Int (−, +), Eng-Ext (−, +), Con-Wst (+, −), and etc. and the next is those changes to the positive side, that is, the relationship between Con and Int varies from (0, 0) in direct to (+, +) in integral, and so does between Ext-Int, Eng-Wst, IBS-Wst, and Wst-Ext. Such result indicates that relationships between compartments are more prone to system-wide stability and benefits.

Then, we tried to analyze each trophic level. There are some differences in respective mutual relations between producers (Int and Ext). Int has a variety of links and its direct and integral relationships are not unified. Both the direct and indirect linkages for Ext mainly indicate that it exploits consumers (Eng, Con, and IBS) in terms of advancing raw materials, apparatus, and energy, and then being exploited by decomposer (Wst) for carbon recycle. As the largest carbon doners, Ext dominates the contacts between producers and other compartments. The irreplaceable effect of decomposer leads to obvious direct relationships that Wst is exploited by first consumer (Con) in dealing with carbon waste and pays back to producer (Ext). Besides, there are no more direct links between other consumers and producer but diverse indirect interactions. First consumers (Eng and Con) play a significant role in transform carbon from distal environment into industrial society, so they mainly exchange carbon with producer (especially Ext) in terms of raw materials and energy. And particularly, Con is exploited by producer and second consumer (IBS) in material import and service management. Located at the highest trophic level, utility consumer (IBS) is mainly exploited by producer (Int and Ext) and first consumers (Eng and Con), for it acts better as a carbon recipient. Yet the direct and integral relationships between IBS and the decomposer (Wst) are in neutrality and mutualistic condition. That is because there is no direct carbon exchange between them, while both of them are exploited by Con as a carbon accepter in indirect ways. In a word, these compartments all play their own role in ecological structure of carbon metabolism in BDA.

### 3.3. Network Control Condition

**Figure 3:**
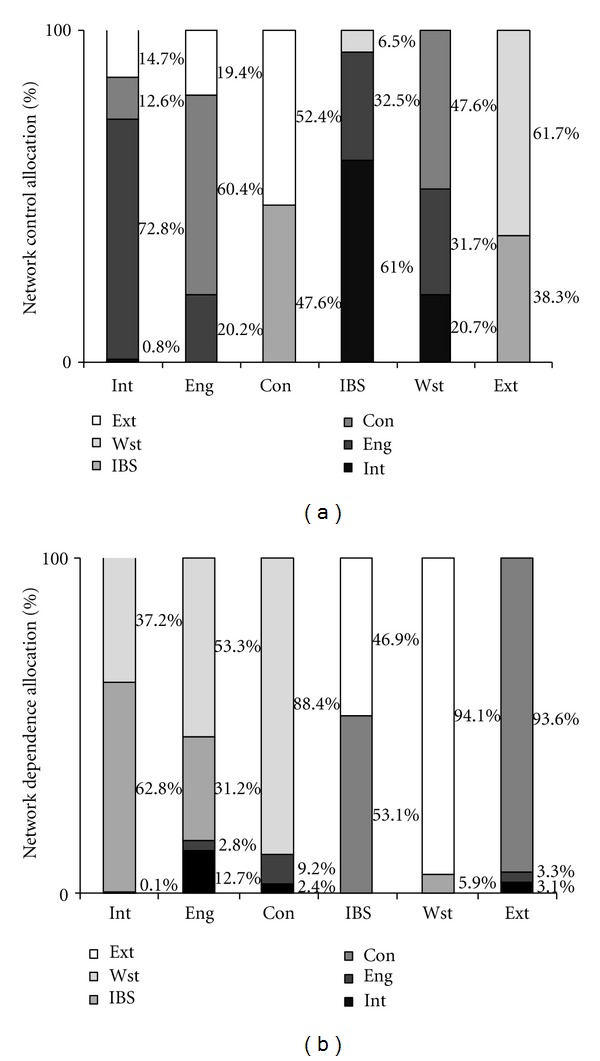
(a) Network control of carbon metabolism in BDA. (b) Network dependence of carbon metabolism in BDA. Because the value of total supplied carbon from Grn is zero, we did not consider the influence from Grn in these figures for an easy calculation.

Figures 3(a) and 3(b) illustrate the proportion for control and dependence condition of LCMN in BDA, respectively. These proportions are extracted from the control allocation matrix (CA) and dependence allocation matrix (DA) introduced in the preceding part, which indicates the control degree based on the controller's output environ and dependence degree from the being controlled input environ.

From the dependent perspective, Eng and Con as first consumers have some differences on dependence degree, and there exists an intrinsic linkage. Eng is more controlled by Con (60.4%), while Con is dependent on second consumer's management (IBS, 47.6%). The second consumer (IBS) generally depends on Eng's energy furnish (32.5%), whereas the influence from Wst (6.3%) is too small to mention. And at the decomposer's level (Wst), the control degree is mostly contributed by first consumers, where Con achieves 47.6% and Eng as 31.7%. The control condition shows a similarity (even more pronounced) in the systemic control for carbon metabolic in the views of controller. In addition, by observing the producers dependence degree, first consumers are more dominated by Ext (Eng is 19.4%, while Con is 52.4%), while second consumer (IBS, 61.0%) and decomposer (Wst, 20.7%) are more controlled by Int. Similar results have also been reflected in the control condition, where the control proportions from Ext to IBS and Wst reach 46.9% and 94.1%, while those from Int to Eng and Con achieve 12.7% and 2.4%. These similar results announce that the BDA International Park is further regulated by the outside world (especially by the external environ), so as to enhance the integral cooperation between those compartments, which may be a promising way to improve the utility of carbon metabolic in BDA.

### 3.4. System Condition

The calculating values of system-wide indicators for carbon metabolism in BDA are all shown in Table 4. Apparently, compared with the carbon flows without internal and external environment, there are more connections among network compartments. Similar results are also shown in the Link Density and Connectance, where suggesting that with cooperation of Int and Ext, there are more diverse cycling ways. These results emphasize the significant affection and higher efficiency of producers (Int and Ext). Moreover, the whole system properties of LCMN in BDA have higher MI (1.50) and SI (3.57), indicating the industrial system holds more positive relationships in an integral way. Due to the fact that Mi is >1.00 both with and without Int and Ext, the carbon metabolic system in BDA can maintain self-mutualism and sustainability despite the lack of external supply. In natural ecosystem, it is quite often the case that more positive utilities than negative ones are needed to keep self-mutualism [31]. Making an analogy with natural ecosystem, these results may evince a sustainable improvement of carbon metabolism in BDA International Business Park.

## 4. Conclusions

Based on the methodology of ENA, we established a metabolic model for low-carbon high-tech industrial parks and analyzed the carbon metabolic system of the BDA International Business Park in this research. The results reflected the behaviors and potential linkage of system compartments and revealed the structure, function, and mutualism condition of the carbon metabolic system in the case park. In the relationship and control analyses, compartments links are quite diverse and positive, especially the Con and Wst, which play a pivotal role in exchanging carbon flows between industrial compartments and environs (both Int and Ext). Comparing the scenarios with Int and Ext or not, variations of system-wide indicators have revealed the significance of artificial control including carbon supplying and decomposing. Regarding ecological structure and function, the pyramidal ecological structure and positive indicators (MI and SI) both show a system mutualism and sustainable condition, which makes BDA Park a demonstration project in Chinese low-carbon EIPs' construction. Yet if we enhanced the effect of waste management part, different system functions would go on better.

## Figures and Tables

**Table 1:** System-wide indicators of metabolism model.

Indicators	Formulation	Short description
Nodes	m	Quantity of metabolic compartments, also the size of network
Links	L	Quantity of metabolic direct flows or arcs
Link density	L/m	Metabolic linking degree
Connectance	L/m^2^	Metabolic connectivity, also the proportion of realized direct pathways
MI	Equation (3)	Metabolic system
SI	Equation (4)	Metabolic system synergism
CI	Equation (9)	Self-regulation of metabolism

**Table 2:** Carbon flows within the low-carbon metabolic network of BDA (unit: t CO_2_-eq)^a^.

Doner/accepter	Int	Eng	Con	IBS	Wst	Grn	Ext	*T*_*j*_
Int	0	3046	0	39	0	0	6014	9100
	(0.0%)	(1.4%)	(0.0%)	(0.0%)	(0.0%)	(0.0%)	(2.8%)	(4.3%)
Eng	0	0	0	0	0	0	12796	12796
	(0.0%)	(0.0%)	(0.0%)	(0.0%)	(0.0%)	(0.0%)	(6.0%)	(6.0%)
Con	13	0	0	6001	0	0	82509	88523
	(0.0%)	(0.0%)	(0.0%)	(2.8%)	(0.0%)	(0.0%)	(38.6%)	(41.4%)
IBS	6001	8288	0	0	0	0	23	14312
	(2.8%)	(3.9%)	(0.0%)	(0.0%)	(0.0%)	(0.0%)	(0.0%)	(6.7%)
Wst	0	0	17547	0	0	0	0	17547
	(0.0%)	(0.0%)	(8.2%)	(0.0%)	(0.0%)	(0.0%)	(0.0%)	(8.2%)
Grn	2176	4644	6408	2234	0	0	36920	52382
	(1.0%)	(2.2%)	(3.0%)	(1.0%)	(0.0%)	(0.0%)	(17.3%)	(24.5%)
Ext	0	1462	0	0	17547	0	0	19009
	(0.0%)	(0.7%)	(0.0%)	(0.0%)	(8.2%)	(0.0%)	(0.0%)	(8.9%)
*T*_*i*_	8190	17441	23955	8274	17547	0	138262	**213670**
	(3.8%)	(8.2%)	(11.2%)	(3.9%)	(8.2%)	(0.0%)	(64.7%)	

^a^The numbers in parentheses mean the proportion of carbon flows, namely, the flow value divided by the total carbon flow in the whole system. *T*_*i*_ is the sum of flows put into the *j*-th compartment, and *T*_*j*_ is for the sum flows into the *j*-th compartment analogously. The *T*_*i*_-*T*_*j*_ intersecting number in bold indicates the total carbon flow of BDA.

**Table 3:** Direct utility sign matrix (Sign *D*)/integral utility sign matrix (Sign*U*) of LCMN in BDA^a^.

	Int	Eng	Con	IBS	Wst	Ext
Int	0/+	+/−	0/+	−/−	0/−	0/+
Eng	−/−	0/+	0/−	−/−	0/+	+/+
Con	0/+	0/−	0/+	+/+	−/−	+/+
IBS	+/+	+/+	−/−	0/+	0/+	0/−
Wst	0/+	0/+	+/+	0/+	0/+	−/+
Ext	−/+	−/−	−/−	0/−	+/+	0/+

^a^Due to the value of total supplied carbon from Grn is zero, as the carbon sink, we can consider that the mutual relationships between other compartments and Grn are exploit-exploited (including Int, Eng, Con, IBS, and Ext) or neutrality (including Wst). In this sense, we did not take into consideration Grn calculation and comparison in Table 3. In addition, the signs of direct and integral utility matrixes for each pair of compartments are separated by “/”, for example, “+/−” and “−/−” between Eng and Int show that the direct mutual relationship between them is (+, −), while the integral interaction is (−, −).

**Table 4:** System-wide indicators of carbon metabolism in BDA.

Indicators	Formulation	Low-carbon metabolic network	
		with Int and Ext	without Int and Ext
Nodes	m	7	5
Links	L	15	6
Link density	L/m	2.14	1.20
Connectance	L/m^2^	0.31	0.24
MI	Equation (3)	1.50	1.40
SI	Equation (4)	3.57	2.54

## References

[1] Doyle B, Martin SA, Weitz KA Eco-industrial parks: a case study and analysis of economic, environmental, technical and regulatory issues.

[2] Zhu Q, Cote RP (2004). Integrating green supply chain management into an embryonic eco-industrial development: a case study of the Guitang Group. *Journal of Cleaner Production*.

[3] Sendra C, Gabarrell X, Vicent T (2007). Material flow analysis adapted to an industrial area. *Journal of Cleaner Production*.

[4] Desrochers P (2004). Industrial symbiosis: the case for market coordination. *Journal of Cleaner Production*.

[5] Allison I, Bindoff NL, Bindschadler RA

[6] IPCC Climate Change 2007, Synthesis Report.

[7] Maes T, Van Eetvelde G, De Ras E (2011). Energy management on industrial parks in Flanders. *Renewable and Sustainable Energy Reviews*.

[8] Roberts BH (2004). The application of industrial ecology principles and planning guidelines for the development of eco-industrial parks: an Australian case study. *Journal of Cleaner Production*.

[9] Hashimoto S, Fujita T, Geng Y, Nagasawa E (2010). Realizing CO_2_ emission reduction through industrial symbiosis: a cement production case study for Kawasaki. *Resources, Conservation and Recycling*.

[10] Lehtoranta S, Nissinen A, Mattila T, Melanen M (2011). Industrial symbiosis and the policy instruments of sustainable consumption and production. *Journal of Cleaner Production*.

[11] Sokka L, Pakarinen S, Melanen M (2011). Industrial symbiosis contributing to more sustainable energy use—an example from the forest industry in Kymenlaakso, Finland. *Journal of Cleaner Production*.

[12] Korhonen J, Wihersaari M, Savolainen I (2001). Industrial ecosystem in the Finnish forest industry: using the material and energy flow model of a forest ecosystem in a forest industry system. *Ecological Economics*.

[13] Liu LX, Zhang B, Bi J, Wei Q, Pan H (2012). The greenhouse gas mitigation of industrial parks in China: a case strudy of Suzhou Industrial Park. *Energy Policy*.

[14] Norstebo VS, Midthun K, Bjorkvoll T (2012). Analysis of carbon capture in an industrial park—a case study. *International Journal of Greenhouse Gas Control*.

[15] Midthun K, Norstebo VS, Perez-Valdes G, Bjorkvoll T (2012). Investment analysis of an integrated industrial park with carbon capture. *Journal of Natural Gas Science and Engineering*.

[16] Munir SM, Manan ZA, Alwi SRW (2012). Holistic carbon planning for industrial parks: a waste-to-resources process integration approach. *Journal of Cleaner Production*.

[17] Tudor T, Adam E, Bates M (2007). Drivers and limitations for the successful development and functioning of EIPs (eco-industrial parks): a literature review. *Ecological Economics*.

[18] Anderberg S (1998). Industrial metabolism and the linkages between economics, ethics and the environment. *Ecological Economics*.

[19] Ayres RU, Simonis UE (1994). *Industrial Metabolism: Restructuring for Sustainable Development*.

[20] Chen L, Wang RS, Yang JX, Shi YL (2010). Structural complexity analysis for industrial ecosystems: a case study on LuBei industrial ecosystem in China. *Ecological Complexity*.

[21] Graedel T, Allenby BR, Shi H (2004). *Industrial Ecology*.

[22] Tian JP, Shi H, Chen Y, Chen LJ (2012). Assessment of industrial metabolisms of sulfur in a Chinese fine chemical industrial park. *Journal of Cleaner Production*.

[23] Fang YP, Zhou HZ (2009). Value flow analysis based on EAP industrial chain: case of Huaning in Xichang, Sichuan. *Journal of Cleaner Production*.

[24] Singh A, Lou HH, Yaws CL, Hopper JR, Pike RW (2007). Environmental impact assessment of different design schemes of an industrial ecosystem. *Resources, Conservation and Recycling*.

[25] Ashton W (2008). Understanding the organization of industrial ecosystems: a social network approach. *Journal of Industrial Ecology*.

[26] Zheng HM, Zhang Y, Yang NJ (2012). Evaluation of an eco-industrial park based on a social network analysis. *Procedia Environment Sciences*.

[27] Fath BD, Patten BC (1998). Network synergism: emergence of positive relations in ecological systems. *Ecological Modelling*.

[28] Shi H, Moriguichi Y, Yang J (2003). Industrial ecology in China, part I: research. *Journal of Industrial Ecology*.

[29] Patten BC, Higashi M, Burns TP (1991). Network ecology: indirect determination of the life-environment relationship in ecosystems. *Theoretical Studies of Ecosystems: The Network Perspective*.

[30] Patten BC (1992). Energy, emergy and environs. *Ecological Modelling*.

[31] Fath BD (2007). Network mutualism: positive community-level relations in ecosystems. *Ecological Modelling*.

[32] Fath BD, Borrett SR (2006). A MATLAB function for network environ analysis. *Environmental Modelling & Software*.

[33] Lobanova G, Fath BD, Rovenskaya E (2009). Exploring simple structural configurations for optimal network mutualism. *Communications in Nonlinear Science and Numerical Simulation*.

[34] Zhang Y, Yang ZF, Yu XY (2009). Ecological network and emergy analysis of urban metabolic systems: model development, and a case study of four Chinese cities. *Ecological Modelling*.

[35] Chen SQ, Chen B (2012). Network environ perspective for urban metabolism and carbon emissions: a case study of Vienna, Austria. *Environmental Science and Technology*.

[36] Patten BC (1978). Systems approach to the concept of environment. *The Ohio Journal of Science*.

[37] Patten BC, Auble GT (1981). System theory of the ecological niche. *American Naturalist*.

[38] Fath BD (2004). Distributed control in ecological networks. *Ecological Modelling*.

[39] Fath BD, Patten BC (1999). Quantifying resource homogenization using network flow analysis. *Ecological Modelling*.

